# Classification of blood pressure during sleep impacts designation of nocturnal nondipping

**DOI:** 10.1371/journal.pdig.0000267

**Published:** 2023-06-13

**Authors:** Bobak J. Mortazavi, Josefa L. Martinez-Brockman, Baylah Tessier-Sherman, Matthew Burg, Mary Miller, Zhale Nowroozilarki, O. Peter Adams, Rohan Maharaj, Cruz M. Nazario, Maxine Nunez, Marcella Nunez-Smith, Erica S. Spatz

**Affiliations:** 1 Department of Computer Science & Engineering, Texas A&M University, College Station, Texas, United States of America; 2 Center for Remote Health Technologies and Systems, Texas A&M University, College Station, Texas, United States of America; 3 Yale/Yale New Haven Health System Corporation Center for Outcomes Research and Evaluation, New Haven, Connecticut, United States of America; 4 Equity Research and Innovation Center, Yale School of Medicine, New Haven, Connecticut, United States of America; 5 Section of Cardiovascular Medicine, Department of Internal Medicine, Yale School of Medicine, New Haven, Connecticut, United States of America; 6 Department of Anesthesiology, Yale School of Medicine, New Haven, Connecticut, United States of America; 7 Department of Family Medicine, Faculty of Medical Sciences, University of the West Indies, Cave Hill, Barbados; 8 Department of Paraclinical Sciences, University of the West Indies, Saint Augustine, Trinidad; 9 Department of Biostatistics and Epidemiology, Graduate School of Public Health, University of Puerto Rico, San Juan, Puerto Rico; 10 School of Nursing, University of the Virgin Islands, US Virgin Islands; 11 Section of General Internal Medicine, Department of Medicine, Yale School of Medicine, New Haven, Connecticut, United States of America; Research Institute of the McGill University Health Centre, McGill University, CANADA

## Abstract

The identification of nocturnal nondipping blood pressure (< 10% drop in mean systolic blood pressure from awake to sleep periods), as captured by ambulatory blood pressure monitoring, is a valuable element of risk prediction for cardiovascular disease, independent of daytime or clinic blood pressure measurements. However, capturing measurements, including determination of wake/sleep periods, is challenging. Accordingly, we sought to evaluate the impact of different definitions and algorithms for defining sleep onset on the classification of nocturnal nondipping. Using approaches based upon participant self-reports, applied definition of a common sleep period (12 am -6 am), manual actigraphy, and automated actigraphy we identified changes to the classification of nocturnal nondipping, and conducted a secondary analysis on the potential impact of an ambulatory blood pressure monitor on sleep. Among 61 participants in the Eastern Caribbean Health Outcomes Research Network hypertension study with complete ambulatory blood pressure monitor and sleep data, the concordance for nocturnal nondipping across methods was 0.54 by Fleiss’ Kappa (depending on the method, 36 to 51 participants classified as having nocturnal nondipping). Sleep quality for participants with dipping versus nondipping was significantly different for total sleep length when wearing the ambulatory blood pressure monitor (shorter sleep duration) versus not (longer sleep duration), although there were no differences in sleep efficiency or disturbances. These findings indicate that consideration of sleep time measurements is critical for interpreting ambulatory blood pressure. As technology advances to detect blood pressure and sleep patterns, further investigation is needed to determine which method should be used for diagnosis, treatment, and future cardiovascular risk.

## Author summary

The identification of nocturnal nondipping hypertension has been shown to be a risk factor for future cardiovascular disease, however, requires the accurate capture of blood pressure measurements during wake and sleep periods. We found that, while some cases were clear cases of nocturnal nondipping hypertension, there were a number of cases in which classification of an individual as having nocturnal nondipping hypertension depended upon the inclusion or exclusion of a few readings based upon sleep status. We tested several classification methods and found only partial concordance for the cases classified as nocturnal nondipping. We noted that existing literature also uses pre-defined periods for sleep, participant self-reports for sleep timing, or automated actigraphy, and conclude that future study design must carefully consider sensors to determine wake versus sleep periods and how to treat blood pressure readings taken during sleep disturbances. In addition, we also found that the ambulatory blood pressure monitor was associated with shorter periods of sleep, which may influence classification of nocturnal nondipping in studies that only capture one night of sleep.

## Introduction

The identification of nocturnal blood pressure (night-time blood pressure [BP] >120/70 mmHg) and nocturnal nondipping BP (<10% drop in average systolic BP during sleep) [1] through ambulatory BP monitoring (ABPM) are valuable elements of risk prediction for cardiovascular disease, independent of daytime BP [2–5]. Nocturnal hypertension and nocturnal nondipping are associated with sleep apnea [6,7], diabetes [8,9], complications in pregnancy [10], left ventricular hypertrophy, end organ damage, and stroke [11–16]. While treatment of nocturnal hypertension and nocturnal nondipping lack outcomes data, appropriate diagnosis and treatment may help to prevent hypertension-related sequelae such as heart failure, kidney disease, and cognitive dysfunction [5]. In 2017 the American Cardiology Association and the American Hypertension Society (ACC/AHA) lowered the cutoff for both daytime hypertension (BP>130/80) and nocturnal hypertension (BP>110/65) [1]. A study of the National Health and Nutrition Examination Survey population estimated that 13.3% of U.S. adults (31.5 million) had isolated masked asleep hypertension (elevated nocturnal BP and non-elevated clinic BP reading), per the ACC/AHA guidelines [17]. As new technology advances to better implement the 2017 ACC/AHA guidelines for blood pressure control through ambulatory monitoring, the level of data granularity needed to properly classify participants with different types of hypertension is not known [1].

Capturing nocturnal BP and the associated diagnoses of hypertension is a challenge. Repetitive cuff inflations can alter sleep quality, which in-turn may increase BP above what is typical for the individual [18]. For some patients, the constant inflation of the cuff throughout the night is not tolerated and they discontinue the test. Ideally, nocturnal BP would be measured across several nights to increase reliability of findings [19], especially as nocturnal measurements taken over a single 48-hour period or two 24-hour measurement periods provide significant additional prognostic value for future cardiovascular events and mortality [15,20].

A second challenge is defining the time stamps for sleep, which may vary depending on whether sleep is self-reported or captured more objectively [21,22]. For example, several studies have used actigraphy data, which use automated algorithms to detect sedentary periods at night that represent sleep [23–25], polysomnography with actigraphy data, that correlate lack of activity with classification from polysomnography to detect sleep [26], and actigraphy data with self-reported sleep data [21,22] to determine periods of sleep and quality of sleep [22–26]. In these studies, awake/sleep periods were determined using different methods, including actigraphy and self-report data [23,25,26] and self-report alone [24]; one study defined sleep as any period between midnight and 5 am that overlapped with actigraphy readings [22]. To date, however, there is no gold standard for how best to define the sleep period, or how to interpret interruptions to sleep due to cuff inflations (sleep quality/disturbances), potentially resulting in under- and over-diagnosis of nocturnal hypertension or nocturnal nondipping, which may impact the ability to prognosticate and prescribe treatment [27]. Actigraphy-based approaches are similar but not well-defined with respect to periods of sleep and appropriate handling of sleep disruptions. Accordingly, we sought to evaluate the impact of different definitions and algorithms for defining sleep onset and duration on the classification of nocturnal nondipping. The primary goal was to determine how best to calculate nocturnal BP through different approaches of calculating BP readings taken during sleep. A secondary goal was to evaluate the potential impact of the ABPM on sleep.

Accordingly, the contributions of this article are as follows:

- We demonstrate the importance in defining an appropriate sleep versus wake reading classification, showing the impact this has on identifying nocturnal non dipping.
- We show that changes in defining sleep versus wake readings can impact diagnosis of hypertension, which can have impact in downstream clinical decision making.
- We identify challenges in defining periods of sleep via actigraphy, in relation to sleep disturbances, and the impact these disturbances and sleep quality have on hypertension classifications.

## Results

### Cohort

**Table 1** presents demographics of these participants as a subset of the larger Eastern Caribbean Health Outcomes Research Network (ECHORN) hypertension study [28]. The ECHORN hypertension sub-project was approved by the Yale University Human Subjects Investigation Committee, and the Institutional Review Boards of the University of Puerto Rico Medical Sciences Campus, the University of the US Virgin Islands, the University of the West Indies, and the Ministry of Health Trinidad and Tobago. Written informed consent was required for participation in the study. The current analyses are covered by the original protocol.

**Table 1:** 61 participant characteristics.

Participant characteristics	N	%
Age, mean (standard deviation)	57.9	(8.51)
Sex		
Female	40	65.57
Male	21	34.43
Site		
Barbados	28	45.90
Puerto Rico	23	37.70
Trinidad and Tobago	1	1.64
United States Virgin Islands	9	14.75
Body Mass Index		
Normal or underweight (< 25)	10	16.39
Overweight (25–29.9)	31	50.82
Obese (> = 30)	20	32.79
Elevated waist to hip ratio*	39	63.93
Current smoking	1	1.64
Current alcohol use	27	44.26
Diabetes**	6	9.84
Cancer	3	4.92
Sleep Apnea	2	3.28

* waist to hip ratio: if gender is male, waist to hip ratio > .9 is used, if female > .85 is used

** Diabetes: self report or A1c > = 6.5% or fasting glucose > 125 or meds

A total of 77 participants were enrolled in the study from July 2018 through November of 2020. We conducted pre-processing of the data to make it ready for the analysis by removing erroneous readings from the ABPM, and excluded participants that had no actigraphy data, or had fewer than 12 ABPM readings. Once our periods of sleep were identified by our four techniques, any participant with no readings during sleep were also removed (perhaps because of only erroneous cuff inflations). Of these 77 participants, 61 were included in our final analysis.

The mean age of participants was 57.9 years (SD 8.5) and the majority were female. Most were enrolled from either Barbados or Puerto Rico. Most participants (83.6%) were overweight or obese and had a high waist to hip ratio (> 0.9 for male, > 0.85 for female). Fewer than 10% had diabetes, <2% smoked, and <5% had a diagnosis of sleep apnea.

Based upon wave 2 study visit BP readings and ABPM readings, **Table 2** shows the types of hypertension identified in participants, including 24.6% with hypertension, 21.0% with white coat hypertension and 4.9% with masked hypertension. There was variation in classification of nocturnal nondipping between manual calculations based upon the method of sleep calculation: 1) self-reported by participants and initialized into the ambulatory blood pressure monitoring (the ABPM was then pre-programed to capture readings every 30 minutes during wake periods and every 60 minutes during sleep periods, hereafter ABPM–self-report sleep/wake), 2) any reading captured between 12 am to 6 am (hereafter ABPM– 12 am to 6 am), and 3) based on actigraphythrough “visual” analysis of sedentary periods (hereafter Actigraphy–manual) and 4) through automated actigraphy using the Cole-Kripke algorithm for sleep/wake and Tudor-Locke for sleep duration calculations (hereafter Actigraphy–automated) [29].

**Table 2:** Hypertension Classification.

Classification	Counts
Normotensive	22
Sustained HTN	15
Masked HTN	3
White Coat HTN	21
Nocturnal nondipping–ABPM—self-report	51
Nocturnal nondipping–ABPM– 12 am to 6 am	36
Nocturnal nondipping–Actigraphy–manual	43
Nocturnal nondipping–Actigraphy—automated*	42

*8 participants were classified as nocturnal nondipping htn with manual actigraphy because they had insufficient persistent sedentary time to qualify as sleep via automated analysis.

### Nocturnal nondipping

**Table 2** quantifies the differences in participants as classified as having nocturnal nondipping by different calculation methods for sleep periods, all using the definition of a difference of 10% between the mean awake and sleep systolic blood pressures (SBPs). For ABPM (self-reports), 51 of the 61 participants were classified as having nocturnal nondipping. Defining sleep time from 12 am to 6 am, 36 participants were classified as having nocturnal nondipping. Defining sleep time by manual processing of actigraphy data resulted in 43 of the participants being classified as having nocturnal nondipping, and 42 using automated actigraphy analysis. Relevant only to the last method of automated analysis, 11 of the 42 participants had insufficient persistent sedentary time to qualify as sleep, and thus, had no recorded sleep time; thus, we used manual assessment of sleep actigraphy (method 3) to classify these participants. Eight of the 11 participants with insufficient sleep were found to have nocturnal nondipping through manual analysis of sleep actigraphy.

We additionally evaluated the concordance of the methods to define nocturnal nondipping by different sleep calculation methods. The self-report data presents the largest number of nocturnal nondipping. Of the 51 participants identified as having nocturnal nondipping from the self-report, 36 participants also qualified using the 12 am to 6 am readings, 41 using manual actigraphy, and 33 using automated actigraphy. The 36 participants identified as having nocturnal nondipping based on 12 am to 6 am BP readings were all considered to have nocturnal nondipping as determined by other approaches. Twenty of the 61 participants had varied classification depending upon the awake versus sleep method used.

Pairwise agreement on nondipping BP classification by the various methods of determining sleep time is reported in **Table 3**. Cohen’s kappa coefficient ranged from 0.44 to 0.65. The mean Cohen’s Kappa was 0.55 across all comparisons. The Fleiss’ Kappa for multi-rater comparisons and the Fleiss Kappa for these methods was 0.54.

**Table 3:** Pairwise concordance testing.

Classification Method	Calculated by readings from ABPM (self-report)	Calculated by readings from ABPM (12am-6am)	Calculated by Actigraphy—manual	Calculated by Actigraphy—automated
Calculated by readings from ABPM (self-report)				
Calculated by readings from ABPM (12am-6am)	0.44			
Calculated by Actigraphy—manual	0.46	0.54		
Calculated by Actigraphy—automated	0.60	0.58	0.65	

### Sleep vs. wake blood pressures for nocturnal nondipping for all classification strategies

For each of the awake and sleep classification strategies, the wave 2 study visit blood pressure, the awake and sleep SBPs from ABPM, and the threshold value for BP dipping during sleep are provided in **Table 4**. The threshold values represent a 10% reduction in SBP from the mean awake SBP. **Table 4** demonstrates similar mean awake SBP readings, no matter the method of sleep calculations, and a difference between mean sleep SBP between dipping and nondipping groups.

**Table 4:** Blood Pressure Calculations/Trends. Values are Mean mmHg (interquartile range).

Nocturnal Nondipping Classification		Resting Clinic BP Readings		ABPM Readings		Nondipping Threshold
		Systolic	Diastolic	Awake Systolic	Sleep Systolic	
Calculated by readings from ABPM (self-report)	Nondipping (n = 51)	132.6 (119.0–146.0)	81.0 (71.5–91.0)	120.6 (111.1–129.1)	116.7 (105.2–125.1)	108.6 (100.0–116.2)
	Dipping (n = 10)	126.1 (115.5–134.5)	74.6 (69.5–82.5)	122.0 (118.8–124.9)	103.7 (95.1–108.9)	109.8 (107.0–112.4)
Calculated by readings from ABPM (12am-6am)	Nondipping (n = 36)	135.0 (120.8–146.0)	83.0 (74.8–93.3)	125.0 (117.7–130.6)	121.0 (114.3–127.8)	112.0 (106.0–117.6)
	Dipping (n = 25)	127.0 (111.0–138.0)	76.0 (67.0–83.0)	122.0 (110.0–129.7)	101.0 (92.3–104.8)	109.7 (99.0–116.7)
Calculated by Actigraphy—manual	Nondipping (n = 43)	134.2 (121.5–146.0)	82.5 (75.0–92.5)	122.8 (112.1–130.4)	117.8 (109.5–125.5)	110.5 (100.9–117.3)
	Dipping (n = 18)	125.0 (109.2–137.5)	73.9 (67.0–82.5)	116.2 (110.7–120.4)	98.2 (94.0–103.6)	104.5 (99.6–108.4)
Calculated by Actigraphy—automated	Nondipping (n = 34)	132.5 (118.8–146.0)	82.1 (75.0–92.8)	120.6 (111.9–129.1)	115.6 (106.9–122.4)	108.5 (100.8–116.2)
	Dipping (n = 27)	130.3 (116.5–142.5)	77.3 (67.5–83.0)	116.7 (107.3–124.6)	99.6 (95.5–103.6)	105.0 (96.6–112.2)

### Sleep quality: changes in sleep measures with and without ABPM

**S**leep efficiency and sleep disturbances were similar among participants classified as nocturnal dippers with participants classified as nocturnal nondippers by each of the methods (**Table 5**). However, the mean awakening lengths differed between nocturnal nondippers classified using automated actigraphy versus self-reported habitual sleep times (**Table 6**).

**Table 5:** Sleep Quality Association with likelihood of nondipping (Mean and IQR) and tests of statistical significance in difference.

Nocturnal Non-Dipping Classification		Total Sleep Time in minutes		Efficiency		Number of Awakenings		Length of Awakenings in minutes	
Calculated by readings from ABPM (self-report)	Nondipping (n = 51)	355.4 (207.2–400.8)	P = 0.4297	90.76 (86.94–95.26)	P = 0.1444	10.45 (5.25–14.75)	P = 0.4658	2.44 (1.82–3.00)	*P = 0.02823
	dipping (n = 10)	382 (225–407)		89.53 (84–95.77)		11.1 (5–16)		2.75 (2.10–3.36)	
Calculated by readings from ABPM (12am-6am)	Nondipping (n = 36)	341.5 (204.5–381.0)	P = 0.1673	90.67 (87.05–94.73)	P = 0.4431	10.12 (6–14)	P = 0.2252	2.46 (1.86–3.09)	P = 0.3242
	dipping (n = 25)	383.7 (217.8–415.8)		90.10 (84.88–95.96)		11.12 (5–16)		2.60 (1.99–3.14)	
Calculated by Actigraphy—manual	Nondipping (n = 43)	352.9 (205.8–398.5)	P = 0.4000	90.52 (86.92–95.02)	P = 0.6550	10.38 (6–14)	P = 0.4562	2.45 (1.83–3.02)	P = 0.1657
	dipping (n = 18)	379 (225–404.5)		90.17 (85.09–95.97)		11.02 (5–16)		2.65 (1.99–3.18)	
Calculated by Actigraphy—automated	Nondipping (n = 34)	348.5 (206.5–397)	P = 0.3328	90.78 (87.12–94.73)	P = 0.2847	10.59 (6–14)	P = 0.8953	2.32 (1.75–2.90)	*P = 0.0026
	dipping (n = 27)	378.2 (214.8–407)		89.98 (84.57–95.96)		10.7 (4.75–16)		2.73 (2–3.37)	

**Table 6:** Sleep Quality Association with likelihood of nondipping (Mean and IQR). Statistical test provided for difference in total sleep time comparing data from the ABPM to data from Actigraphy? The remaining columns were not found to have statistically significant differences.

Classification	Total Sleep Time (with ABPM) in minutes	Efficiency (with ABPM)	Number of Awakenings (with ABPM)	Length of Awakenings In min (with ABPM) in minutes	Total Sleep Time (without ABPM) in minutes	Efficiency (without ABPM)	Number of Awakenings (without ABPM)	Length of Awakenings In min (without ABPM) In minutes	Statistical Significance in Difference of Sleep Times
Nocturnal nondipping–ABPM—self-report	283.1 (193–380.5)	90.08 (86.44–93.31)	11.04 (6–15)	2.56 (2.1–3)	380.3 (210.0–408.0)	90.75 (86.93–95.26)	10.83 (6–14)	2.33 (1.75–2.88)	*p = 0.001
Nocturnal nondipping–ABPM– 12 am to 6 am	268.9 (194.0–351.8)	90.82 (88.14–94.19)	9.64 (6.0–13.25)	2.51 (2.03–3.02)	372.3 (206.5–393.2)	90.65 (87.01–94.66)	10.43 (6–14)	2.41 (1.79–3.11)	*p = 0.003
Nocturnal nondipping–Actigraphy–manual	283.4 (190.0–392)	90.06 (87.01–93.24)	10.76 (6–15)	2.54 (2.13–3.07)	382.9 (207.5–413.0)	90.43 (86.92–94.63)	10.88 (6–14)	2.39 (1.77–2.91)	*p = 0.003
Nocturnal nondipping–Actigraphy—automated	281.7 (193.0–387.5)	90.57 (87.49–94.39)	10.41 (6–15)	2.5 (2.12–3)	373.6 (213.8–423)	90.87 (87.11–94.75)	10.66 (6–14)	2.26 (1.72–2.77)	*p = 0.005

Sleep time for participations with nondipping BP was shorter during the nights when the ABPM was worn compared to the nights without the ABPM (**Table 6**). Sleep duration reduction ranged from a mean of 92 to 103 minutes for the the four groups of nondippers (*p* <0.01). Additional detail on sleep is provided in **S2 Text**.

## Discussion

In this study, we sought to determine the effect of different sleep calculation methods on the determination of nocturnal nondipping, as captured by ABPM. Because of a lack of gold standard in establishing sleep versus wake readings, it may be difficult to properly ascertain a status of nocturnal nondippnig. We found that the prevalence of nondipping ranged across the methods of awake versus sleep classification. The automated actigraphy analysis found a low of 61% of participants having nocturnal nondipping whereas the self-reported habitual sleep times had a high of 84% of participants having nocturnal nondipping.

Identifying nocturnal nondipping hypertension with ABPMs is challenging given the difficulty in establishing reliable sleep onset and quality. As can be seen, while the approaches have overlap, there are significant differences in the number of participants classified as having nocturnal nondipping: 1) 83.6% via self-report, 2) 59.0% via readings from 12am-6am, 3) 70.5% via manual actigraphy analysis, and 4) 68.8% via automated actigraphy analysis. Additionally, our secondary analysis found differences in total sleep time on the night the ABPM was worn versus the two nights it was not worn in the participants classified as having nocturnal nondipping. This shorter duration of sleep may be a contributing factor to nocturnal nondipping in this cohort.

While Actigraphy would seem to be the solution, and the increasing prevalence of wearable sensors that track actigraphy makes the capture of these data relevant, appropriate modeling of sleep is essential for using the data from ambulatory blood pressure monitors to interpret blood pressure. These wearable sensing devices, such as commercially available smartwatches and smart rings, for example Oura smart rings and Samsung Gear watches show significant correlation to medically-approved actigraphy devices [30] as well as comparison of such devices (and more) have been made to medically-approved sleep monitoring, to capture sleep staging and sleep quality [31]. The distance from the threshold values that mark nondipping for each of the analyses suggest all techniques evaluated are good at separating most cases of nondipping vs. dipping, but varied across approaches. As a result, differences in classification of nondipping hypertension come from BPs that are measured right near that threshold of sleep/wake periods, as described in the **S1 Text and S1 Table**; i.e., the inclusion or exclusion of a few BPs may drastically change classification of hypertension phenotypes. Identifying individuals that vary based upon the inclusion or exclusion of a few BPs may be illustrative for diagnostic purposes, as well. The appropriate diagnosis of hypertension is essential for early diagnosis and treatment, which is critical to preventing progression to advanced stages of cardiovascular disorders [32]. Further, such early diagnosis with wearable sensing may provide higher quality of care with more personalized treatment process, particularly for under-represented individuals in rural areas far from clinical to diagnosis [33]. In this study we found that Actigraphy provided an interpretation of awake BPs versus sleep BPs, but still left an important open question about how to classify BP measurements taken during sleep disturbances or right at the beginning or ending of sleep periods.

These data are important for researchers and clinicians who may be using different modalities for assessing sleep time and quality to contextualize blood pressure and identify patients with nocturnal hypertension. At the clinical level, the readings can be adjudicated for an individual, however, when aggregating data across participants in a research study, it can be challenging to develop standardized rules that respect the participant-reported sleep times.

Prior studies assessing BP patterns used different methods for defining sleep periods, with some using multiple methods. Two studies defined the sleep period based on participant self-report and confirmed with actigraphy data [23,26]. One study defined the sleep period by actigraphy, supplemented by diary reports [21]. Two studies defined sleep solely by self-report [24,25]. Notably, one study defined windows of “daytime” (7am-10pm) and “nighttime” (12am-5am) BP. The investigators then operationalized “asleep blood pressure” as readings occurring during a period of time that included at least 90% actigraphic sleep. Some of these studies also investigated sleep duration and sleep quality. Three defined sleep duration solely by actigraphy data [23–25]. One study defined sleep duration by actigraphy, supplemented by self-report [21]. One study examined sleep duration by both actigraphy and polysomnography, separately [26], which is often infeasible due to the difficulty in obtaining polysomnography data in more natural environments where ABPMs are intended to capture blood pressure readings. One study combined self-report and actigraphy data to assess sleep duration, finding a moderately significant correlation between sleep diaries and actigraphy [22]. These studies also utilized at least one measure of sleep quality, two of which included measures of self-reported sleep quality alongside measures derived from actigraphy data [25,26]. The remaining three studies examined sleep quality measures defined solely by actigraphy data [22–24]. In our study, actigraphy measurements revealed shorter sleep periods than self-reported sleep hours, which correlates with the finding that individuals slept fewer hours while wearning the ABPM. Still, questions remain about how to adjudicate sleep disturbances with sleep/wake classification, and how to account for nightly variability in sleep and ABPM data. BP devices that are non-disruptive (e.g., cuffless BP devices) are in development and may ultimately overcome these challenges.

### Limitations

There are several key limitations to our study. First, the threshold selected for sedentary motion and sleep classification was set to remove potential disturbances during sleep. However, if such a disturbance occurred at the time of blood pressure measurement then (either as a result of the device itself waking the participant or coincidence of the participant waking up and the cuff inflating), the algorithm assigned these BPs to sleep, which may not be clinically relevant. Second, the actigraphy monitor used in this study did not include a sensor for ambient light. This sensor is often considered important in improving the accuracy of sleep/wake periods by identifying when people typically turn off the light to go to sleep. Additionally, there are more accurate ways to calculate sleep. While a number of wearable sensor approaches are able to assess sleep, through capture of light, heart rate, activity, temperature, and a variety of biomarkers, some may find self-report sufficient while others may require polysomnography to identify periods of sleep. Further investigation is needed, as shown by the differences in nocturnal nondipping classification identified in this work. Finally, the original study was powered for detecting classes of hypertension and not to identify periods of sleep from actigraphy. These analyses should be repeated in larger cohorts using a variety of sleep detection methods with appropriate statistical power to further validate these results.

### Conclusion

The proper identification of sleep is important in the classification of nocturnal blood pressure, which in turn drives the diagnosis of nocturnal nondipping, a condition associated with future cardiovascular risk. Depending upon the classification method, however, This study demonstrated four ways to calculate nocturnal BP by identifying methods by which data can be classified as sleep versus awake BP. The simplest form (all readings between 12 am to 6 am) resulted in 36 of 61 participants classified as nocturnal nondipping, while the user self-reported periods of sleep had 51 nondipping hypertensive classifications. The actigraph-based findings, which were hypothesized to provide the most accurate identification of sleep, resulted in 43 participants being classified as nocturnal nondipping by manual analysis, or 42 by automated analysis. The findings in this work indicate that differenet measurement of sleep and wake periods can impact the diagnosis and ultimately treatment of nocturnal hypertension. In all cases, differences between the nondipping and other participants existed, but there are participants where a single reading included or excluded in the calculation of nocturnal BP can alter the diagnosis. Future investigtation should focus on less disruptive BP measurement so that sleep quality on the night of measurement is not a factor, as well as more nuanced phenotyping of BP and sleep patterns and their association with clinical outcomes.

## Methods

### Study design

For the current analyses we used data from the ECHORN study, designed to identify factors influencing ambulatory BP measurements and identify phenotypes of BP patterns based on sleep, activity, momentary experiences and exposures, and chronic measures of stress; a full description of study methods is provided elsewhere [34]. Briefly, the ECHORN hypertension study uses a sub-sample of adults participating in the ECHORN Cohort Study (ECS), a longitudinal prospective cohort study of community dwelling adults living on the islands of Barbados, Puerto Rico, Trinidad and Tobago, and the U.S. Virgin Islands. The ECHORN hypertension sub-project was approved by the Yale University Human Subjects Investigation Committee, and the Institutional Review Boards of the University of Puerto Rico Medical Sciences Campus, the University of the US Virgin Islands, the University of the West Indies, and the Ministry of Health Trinidad and Tobago. Informed consent was required for participation in the study. The current analyses is covered by the original protocol.

Inclusion criteria for the hypertension study included 1) completed ECS Wave 2 assessment; 2) not taking any anti-hypertensive medication for any reason; 3) have a resting clinic SBP <180 mm Hg or a diastolic blood pressure (DBP) <110 mm Hg. Hypertension was defined in Wave 2 participants if the SBP was greater > = 130 mm Hg or DBP > = 80 mm Hg in either study visit readings or in ambulatory BP measurement averages, as previously described [34]. Nocturnal nondipping was defined as having less than a 10% drop in systolic BP from mean awake to mean sleep readings [35].

Participants had their resting BP measured during a baseline assessment [34], were fitted with a Spacelabs ONTRAK ABP MONITOR (Spacelabs Healthcare, Snoqualmie, WA) for 24-hour BP monitoring. The research team asked each participant “What time do you typically go to bed each night?” and “What time do you typically wake up each morning?” The monitor was pre-programmed with these sleep and wake times so that the cuff inflated every 30 minutes during waking hours and every 60 minutes during sleep. Participants were also asked to wear an Actigraph GT9X-BT for physical activity monitoring and sleep tracking. The Actigraph smartwatch was worn for three nights, one of which was the night the ABPM was also worn. The Actigraph provided data in activity counts along three axes. The vector magnitude of these three axes provides data on the intensity of physical motion, sampling once per minute. We excluded any participant with fewer than 12 BP readings.

**Fig 1:**
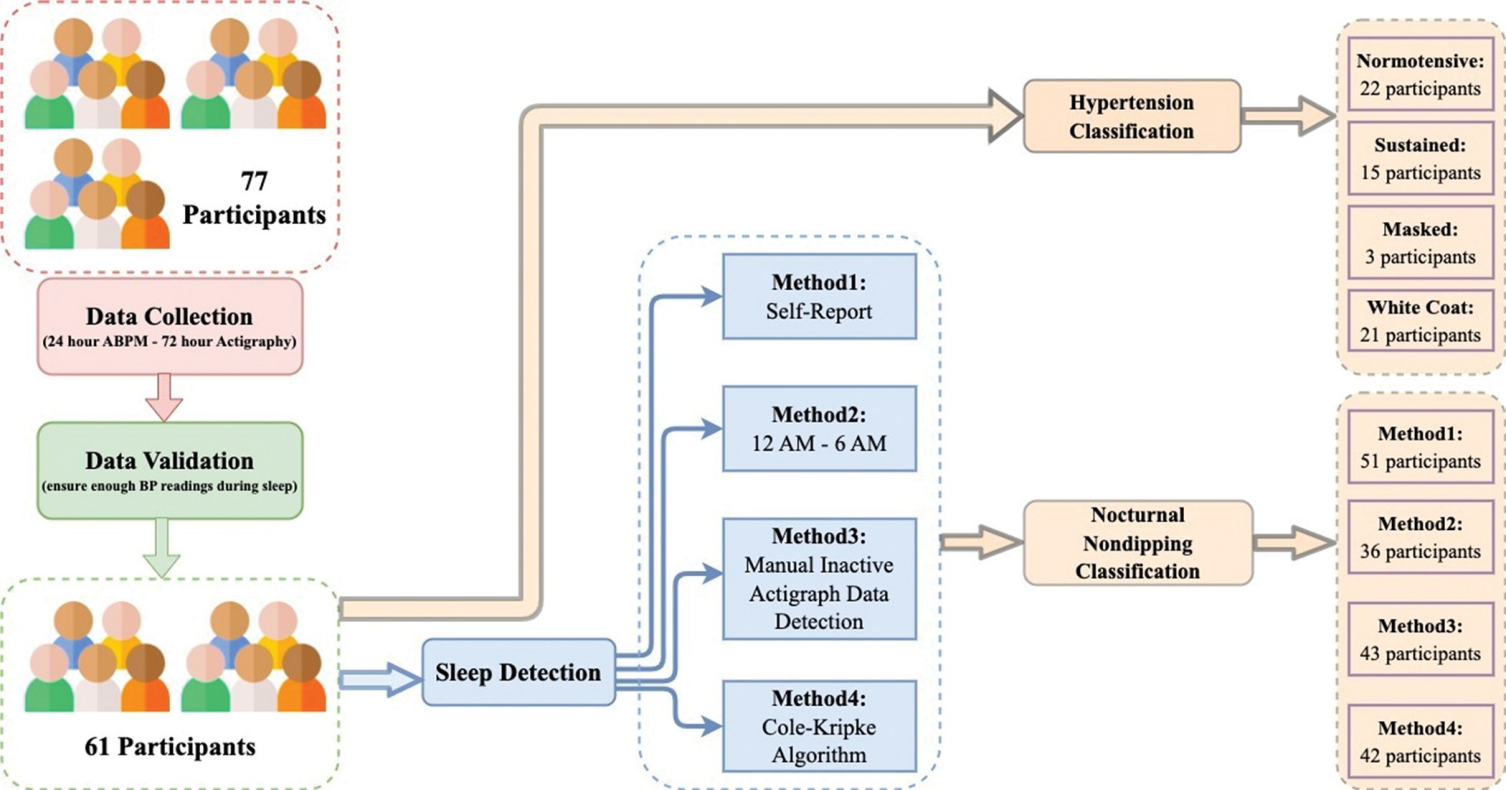
Experimental protocol flow-chart, showing the original data cohort of 77 participants that collected 24 hours of ABPM data and 72 hours of actigraphy data. From this, those with sufficient data were kept and from this four approaches to classifying sleep versus wake readings were conducted.

We analyzed awake versus sleep BP through four different approaches. The first approach used the times by which the ABPM was set to switch from measuring BP every 30 minutes while awake and to every 60 minutes while asleep–as reported by the participants. The second approach defined sleep readings as any reading captured between 12 am and 6 am. The third and fourth approaches concerned capturing periods of sleep through Actigraphy. In the third approach, a period of sleep was defined as the longest subsequence of minimal movement, as captured by Actigraphy. In the fourth approach, the calculations for sleep were based upon the Cole-Kripke algorithm for sleep/wake and Tudor-Locke for sleep duration calculations, as defined through automated analysis [29]. The third and fourth approaches were set to evaluate the difference between what can be calculated “visually” (by looking at data from a device and making a determination) versus what the device, with use of an automated algorithm, defines as sleep. Additional visualizations and comparisons of sleep and wake readings, including removing the first and last sleep reading (as readings that may be as a participant falls asleep and as a participant wakes up) is presented in **S1 Text and S2 Text**. This processing method is illustrated in **Fig 1.**

We also evaluated the reported sleep quality metrics provided by the automated actigraphy analysis. We compared the total sleep time (in minutes), sleep efficiency, number of sleep disturbances, and average length of disturbances (in minutes) for the night the ABPM was worn compared with the two nights it was not worn. While many factors influence sleep quality, this comparison can give some indication of whether wearing the ABPM impacted sleep quality.

### Devices used

Participants were fitted with a Spacelabs ONTRAK ABP MONITOR (Spacelabs Healthcare, Snoqualmie, WA) for 24-hour BP monitoring and were also asked to wear an Actigraph GT9X-BT for physical activity monitoring and sleep tracking. The ABPM captures, for every reading, a systolic blood pressure, a diastolic blood pressure, and heart rate, as well as a status code on whether the reading was captured automatically, manually, or on device retry. The Actigraph captures motion along three axes and calculates a vector magnitude of motion from these directions. It also estimates the number of steps taken, the periods of sitting, standing, and lying, and can take readings of ambient light but this was not used in our study.

### Cohort

**Table 1** presents demographics of these participants as a subset of the larger Eastern Caribbean Health Outcomes Research Network (ECHORN) hypertension study [28]. The ECHORN hypertension sub-project was approved by the Yale University Human Subjects Investigation Committee, and the Institutional Review Boards of the University of Puerto Rico Medical Sciences Campus, the University of the US Virgin Islands, the University of the West Indies, and the Ministry of Health Trinidad and Tobago. Written informed consent was required for participation in the study. The current analyses is covered by the original protocol. A total of 77 participants were enrolled in the study from July 2018 through November of 2020. Of these 77 participants, 61 were included after removing participants who had insufficient data (e.g., removed the actigraph device, had no BP readings during sleep once a period of sleep was identified). The mean age of participants was 57.9 years (SD 8.5) and the majority were female. Most were enrolled from either Barbados or Puerto Rico. Most participants (83.6%) were overweight or obese, and had a high waist to hip ratio. Fewer than 10% had diabetes, <2% smoked, and <5% had a diagnosis of sleep apnea. The final data gathered are described in **Table 7,** which identifies the number of readings, the number of successful readings, and quantity of actigraphy data gathered.

**Table 7:** Dataset Description of the ABPM data and Actigraphy data.

Device	Total number of readings	Number of successful readings	Description of data table columns for analysis
ABPM	9221	6638	Participant key, site identification key, recording date and time, systolic blood pressure, diastolic blood pressure, mean arterial pressure, heart rate, pulse pressure, coefficient of variability, error status, error code, reading status, comment, diary event status, storage of min and max validity ranges, device model, device serial number
Actigraphy	1337514	--	Participant key, site identification key, recording date and time, epoch length in seconds, serial number, axis 1 readings, axis 2 readings, axis 3 readings, steps, vector magnitude, incline off, incline sitting, incline standing, incline lying, dominance

* All actigraph readings are considered successful as there are no error codes associated.

### Defining sleep time by ABPM—Self-report sleep/wake

The first approach used to identify nocturnal BP was to capture this from the ABPM directly. The device switched from capturing readings every 30 minutes to capturing readings every 60 minutes for sleep, based upon participant self-report of habitual sleep and wake time. However, this self-report time was not available to us, so it was inferred from the ABPM directly. We identified nocturnal BP measurements through a three step data processing algorithm. First, we filtered all error codes, saving only captured readings. Second, we identified all readings that were at least 60 minutes apart from each other. Third, we took the longest sequence of these readings and considered these the sleep readings. This involved manually including the last reading, since the gap between the last sleep BP and the next BP reading would be 30 minutes.

### Defining sleep time from ABPM—12 am to 6 am

The second approach to identifying nocturnal BP was to designate nocturnal readings as those between 12 am and 6 am. We chose this window of time to align with prior studies that used an early morning window. Those studies used a combination of early morning window (e.g., 12am-5am) and a period of inactivity as measured by actigraphy [22]. Calculating nocturnal BP readings by this method, we remove the need for participant self-report data and consideration of sleep disturbances that may delay sleep onset. This technique is simple and easy to implement, though subject to reliability concerns when using only a single evening for measurement.

### Defining sleep time by Actigraphy—Manual

The final two approaches to identify sleep and wake times used data from the Actigraph watch. The participants wore the Actigraph watch for 3 days. The approach based upon actigraphy considered the motion data captured only on the day the participants also wore the ABPM for identification of sleep. The manual approach identified the longest consecutive period of inactive data to identify sleep.

To identify the longest consecutive period of inactive data, we used sliding windows of 30 minute readings with 15 minute overlaps from prior windows. These windows provided a moving average filter on the motion across the vector magnitude of the Actigraphy data. If this activity level fell in the bottom tertile of each participant’s total activity across periods of wake or sleep (bottom 33% of activity values), it was considered sedentary; the longest sequence of sedentary behavior was considered sleep.

### Defining sleep time by Actigraphy—Automated

The fourth approach used the Actigraph’s automated calculations of sleep, based upon the Cole-Kripke algorithm for sleep/wake and Tudor-Locke for sleep duration calculations. For this approach, sleep readings were any reading between the calculated “in-bed” and “out of bed” times captured by the device [29,36].

### Identifying sleep and sleep disruptions with Actigraphy

Finally, we looked at the reported sleep quality metrics provided by the automated actigraphy analysis. We compared the total sleep time (in minutes), sleep efficiency, number of sleep disturbances, and average length of disturbances (in minutes) for the night the ambulatory blood pressure monitor was worn compared to the two nights it was not. The measures of sleep quality included efficiency–which captures a representation of the total time in asleep versus the total in bed (potentially awake or asleep) [37,38], the total sleep time, the number of awakenings, the length of those awakenings, and wake after sleep onset. These measures were captured by the automated actigraphy analysis, which determines sleep/wake identification based upon the Cole-Kripke algorithm that ActiLife software uses [36]. We compared these statistics for participants with and without nocturnal nondipping and for nights with and without the ABPM.

### Statistical analysis and software packages

Concordance amongst the different sleep method approaches was calculated using Cohen’s Kappa for pairwise method comparisons and Fleiss’ Kappa for multiple raters interrater reliability. For the sleep analysis, significance testing was conducted to evaluate the differences in total sleep time, efficiency, number of awakenings and length of awakenings. These values (minutes for all time measurements and an efficiency score for efficiency) were continuous value numbers compared with a Welch two sample t-test. We did not control for confounding as this was an exploratory analysis. All analysis was conducted with R 4.1.1, including package vcd (version 1.49) for Cohen’s Kappa and package irr (version 0.84.1) for Fleiss’ Kappa.

## Supporting information

S1 Text Text describing additional inclusion criteria and inclusion/exclusion of readings around sleep. (DOCX)

S2 Text Text describing additional analyses of sleep and sleep disturbances. (DOCX)

S1 Table Classification of nocturnal nondipping after removing first and last sleep readings. (DOCX)

S1 Fig Ambulatory blood pressure monitor readings for a single study participant: Readings from 12 am to 6 am. Red points are considered awake readings. Blue points are considered sleep readings based upon hourly gaps in data. The 12 am to 6 am window is delineated by vertical lines.
(TIFF)

S2 Fig Actigraphy for participant in S1 Fig. (TIFF)

S3 Fig Manual Actigraphy-based sleep analysis. (TIFF)

S4 Fig Ambulatory blood pressure readings illustrated in S1 Fig but with sleep points (blue) indicated by manual actigraphy. (TIFF)

S5 Fig Ambulatory blood pressure readings illustrated in S1 Fig but with sleep points (blue) indicated by automated actigraphy. (TIFF)

S6 Fig Illustrative example of a participant classified as nocturnal nondipping hypertension with manual calculation of self-reported sleep times but not by actigraphy. **S6A Fig** shows the ambulatory blood pressure monitoring data. **S6B Fig** shows the actigraphy analysis that shows the participant woke up, and while remained sedentary for a while after, was deemed to be awake and **S6C Fig** shows the recalculated nocturnal blood pressure with manual actigraphy analysis and **S6D Fig** with automated actigraphy analysis.
(TIF)

S7 Fig Illustrative example of a participant classified as nocturnal nondipping hypertension with actigraphy but not the other methods. **S7A Fig** shows the ambulatory blood pressure monitoring data. **S7B Fig** shows the actigraphy analysis that shows the participant woke up, and while remained sedentary for a while after, was deemed to be awake, and **S7C Fig** shows the recalculated nocturnal blood pressure with manual actigraphy analysis and **S7D Fig** with automated actigraphy analysis.
(TIF)
