# Supplementary material for: Classification of blood pressure during sleep impacts designation of nocturnal nondipping
Source: PLOS Digit Health. 2023 Jun 13;2(6):e0000267. doi: 10.1371/journal.pdig.0000267 (PMC10263317; doi:10.1371/journal.pdig.0000267)
Supplement: S1 Text — (DOCX) [file pdig.0000267.s001.docx]

*Inclusion/Exclusion Criteria based upon number of ambulatory blood pressure monitor readings.*

While traditionally, only readings with at least 8 wake and 4 sleep readings are included for analysis, because this study focused on the definition of sleep readings, any participant with $\geq$ 12 readings was included, without prior consideration of wake/sleep determination. We compared the designation of blood pressure values to awake/sleep times for the following groups: self-reported sleep times as programmed into the ambulatory blood pressure monitor; a standard calculation of sleep time (using any readings between 12 am and 6 am); and Actigraph-inferred sleep (calculated via two different sleep algorithms); each method is described below.

*Inclusion/Exclusion of First and Last Sleep Reading*

An alternative approach was also used that took the same sequence of sleep readings and eliminated the first and last, in case they encompassed a waking period just prior to sleep and just after waking up for the self-report readings and the actigraphy readings. As can be see in **S1 Table**, only 46 participants were classified as having nocturnal nondipping using self-report (as opposed to 51) and 42 using actigraphy (as opposed to 43).
