# Supplementary material for: Classification of blood pressure during sleep impacts designation of nocturnal nondipping
Source: PLOS Digit Health. 2023 Jun 13;2(6):e0000267. doi: 10.1371/journal.pdig.0000267 (PMC10263317; doi:10.1371/journal.pdig.0000267)
Supplement: S2 Text — (DOCX) [file pdig.0000267.s002.docx]

*Sleep Disturbances: Manual vs. Automated Actigraphy*

For both the manual identification of sleep periods, and the automated algorithm provided by Actigraph, the primary concern was to identify sleep disturbances and determine how to classify readings that occurred during these disturbances. For manual identification, we took a conservative approach, capturing sleep readings as only those within the longest continuous sub-sequence of this data. We found tertiles to be robust enough at generating long sequences without potential (small) sleep disturbances affecting sleep calculations of continued sleep. A lower rate (quartile instead of tertile, for example) would often result in a sleep disturbance ending period of sleep prematurely. This threshold was selected to allow for minimal disturbances (such as getting up to use the bathroom). However, a longer period of activity would end the determination of the sleeping period. This means a long period of activity in the middle of two equally lengthened periods of sleep would result in only one being classified as sleep.

Because the manual approach to calculating sleep from actigraphy noted here may prematurely end sleep calculations if a long disturbance occurs during sleep, we also evaluated sleep as calculated by the algorithms provided by Actigraph. Actigraph’s algorithms for sleep similarly seek to capture the longest periods of sleep and sleep disturbances by identifying periods of sleep along with periods of in-bed and out-of-bed. Using their automated approach, we were able to identify longer periods of sleep by allowing for longer sleep disturbances. These algorithms allow for additional sleep disturbances in capturing a sleeping period. If two distinct sleep/wake periods are found in a single night, any reading captured during these periods is considered a sleep reading. We classified based upon both approaches in order to quantify the impact sleep disturbances had on nocturnal blood pressure calculations.

*Case Study: Changes in Sleep BP Calculations for a Single Participant by Different Analysis Methods*

**S1 Fig** selects a single participant’s ambulatory blood pressure monitor readings (chosen at random for illustrative purposes), with the red points considered awake blood pressure and the blue points consider sleep readings based upon the ambulatory blood pressure monitor, with 12 am to 6 am delineated. **S2 Fig** illustrates the actigraphy data for this participant. As can be seen, in the evening period prior to midnight, the participant’s activity winds down, with some minimal disturbance around midnight. The activity remains minimal but present from midnight to 5 am, accounting for movement during sleep and minimal sleep disturbances. However, the activity resumes significantly prior to 6 am. **S3 Fig** illustrates the period of time the manual actigraphy analysis determines is sleep, **S4 Fig** illustrates the ambulatory blood pressure readings as classified by the manual actigraphy analysis and **S5 Fig** by the automated actigraphy analysis. Of note are the three readings included in the automated analysis and not the manual analysis because of a significant sleep disturbance that occurs just prior to midnight (with associated raise in blood pressure).

This subject changes classification from no nocturnal nondipping with the manual analysis, to consideration of nocturnal nondipping because of analysis on the ambulatory blood pressure monitor and manual actigraphy, but not considered nocturnal nondipping with automated actigraphy. This illustrates the difficulty in determining the sleep/wake period for readings and the impact sleep disturbances have on this determination and associated nocturnal nondipping analysis.

*Sleep Calculations using self-reported sleep times programed into the Ambulatory Blood Pressure Monitor*

Of the 61 participants analyzed in the study, 51 were considered to have nocturnal nondipping hypertension when using the sleep data from the ambulatory blood pressure monitor recorded based upon participant self-reports (or 46 with the more strict definition of inclusive points). 10 of these 51 participants were not classified as nocturnal nondipping hypertension when using the actigraphy analysis (or 9 of the 46 when considering the stricter definition in both self-reported and actigraphy cases).

*Sleep Calculations using 12am-6am readings*

By using a standard threshold for sleep time of 12am to 6am, regardless of the self-reported sleep times or actigraphy data, 36 participants were defined as having nocturnal nondipping hypertension. The 36 participants identified as having nocturnal nondipping by manual analysis are all considered to have nocturnal nondipping as calculated by self-reported sleep times in the ambulatory blood pressure monitor and actigraphy. 8 participants identified as having nocturnal nondipping hypertension by manual calculation did not meet criteria for nocturnal nondipping hypertension as calculated by automated actigraphy and 3 if using the manual actigraphy. An example of a participant that changes classification because of actigraphy analysis is provided in **S6 Fig.**

*Sleep Calculations using Actigraph Readings*

Of the 61 participants analyzed in the study, 43 were considered to have nocturnal nondipping hypertension when manually calculating sleep based upon sedentary periods of actigraphy data (42 when using the stricter definition of eliminating the first and last point), and 34 when using the automated algorithm. Of note, 11 participants (of the 61) were deemed to have no sleep/wake time by automated analysis. For these 11 participants, 8 are considered to have nocturnal nondipping hypertension when manually calculating sleep based upon sedentary periods of actigraphy data. There are two participants deemed to have nocturnal nondipping hypertension as calculated by manual actigraphy analysis but not by the other methods, one of which is illustrated in **S7 Fig.**

*Discussion*

The example illustrated in **S1 Fig and S6 Fig** are illuminating when presented with the challenge of identifying nocturnal nondipping hypertension in a scenario where the data is relatively clean. Clearly, the self-report data and actigraphy suggest different periods of sleep as well as sedentary behavior. However, the key difference appears to lie in the period of data just before falling asleep, and this similarly alters classification as illustrated in **S7 Fig**. This participant, in **S7 Fig,** is particularly illustrative because the actigraphy does indicate a reduction in motion in earlier evening, but a sleep disturbance modifies the manual approach to calculating sleep blood pressure. Similarly, the automated approach captures a wider array of points that likely include periods in bed but not yet asleep. In both cases (the exclusion of low blood pressures in the first case and inclusion of high blood pressures in the second case) make it difficult to ascertain whether the individual is asleep and has extremely restless sleep or is just sedentary and not yet asleep, and whether these points should be considered sleep or wake blood pressure readings. The self-reported sleep time does not include readings that the actigraphy monitor does, thus changing the classification of sleep, and vice-versa.
