# Supplementary material for: Classification of blood pressure during sleep impacts designation of nocturnal nondipping
Source: PLOS Digit Health. 2023 Jun 13;2(6):e0000267. doi: 10.1371/journal.pdig.0000267 (PMC10263317; doi:10.1371/journal.pdig.0000267)
Supplement: S1 Table — (DOCX) [file pdig.0000267.s003.docx]

**Supplemental Table 1.** Classification of nocturnal nondipping after removing first and last sleep readings.

| Nocturnal nondipping – ABPM - self-report after removing first and last “sleep” readings | 46 |
| --- | --- |
| Nocturnal nondipping – Actigraphy – manual after removing first and last “sleep” readings | 42 |
