# Supplementary figures and images for: Classification of blood pressure during sleep impacts designation of nocturnal nondipping
Source: PLOS Digit Health. 2023 Jun 13;2(6):e0000267. doi: 10.1371/journal.pdig.0000267 (PMC10263317; doi:10.1371/journal.pdig.0000267)

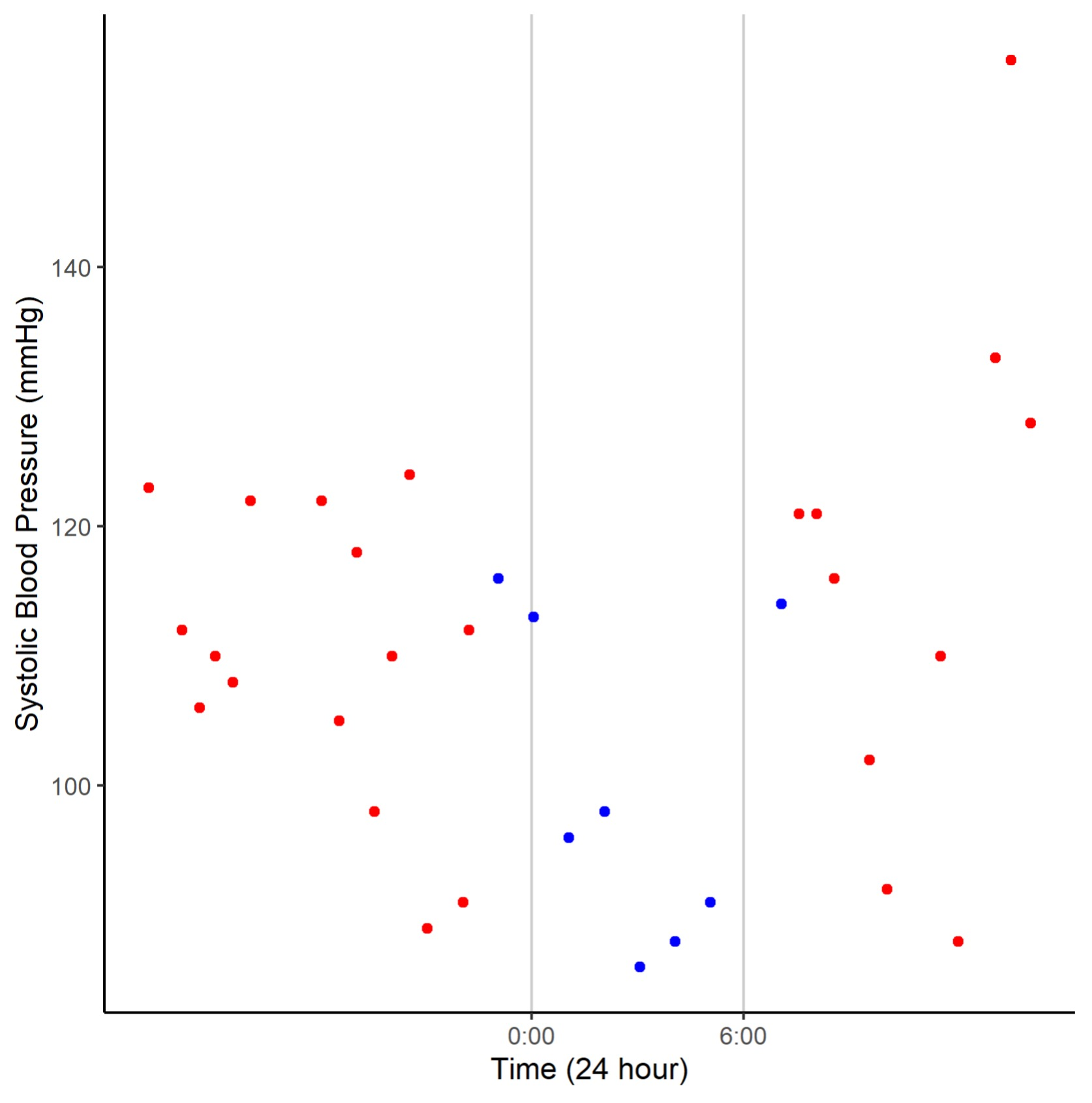

Supplement: S1 Fig — Red points are considered awake readings. Blue points are considered sleep readings based upon hourly gaps in data. The 12 am to 6 am window is delineated by vertical lines. (TIFF) [file pdig.0000267.s004.tiff]

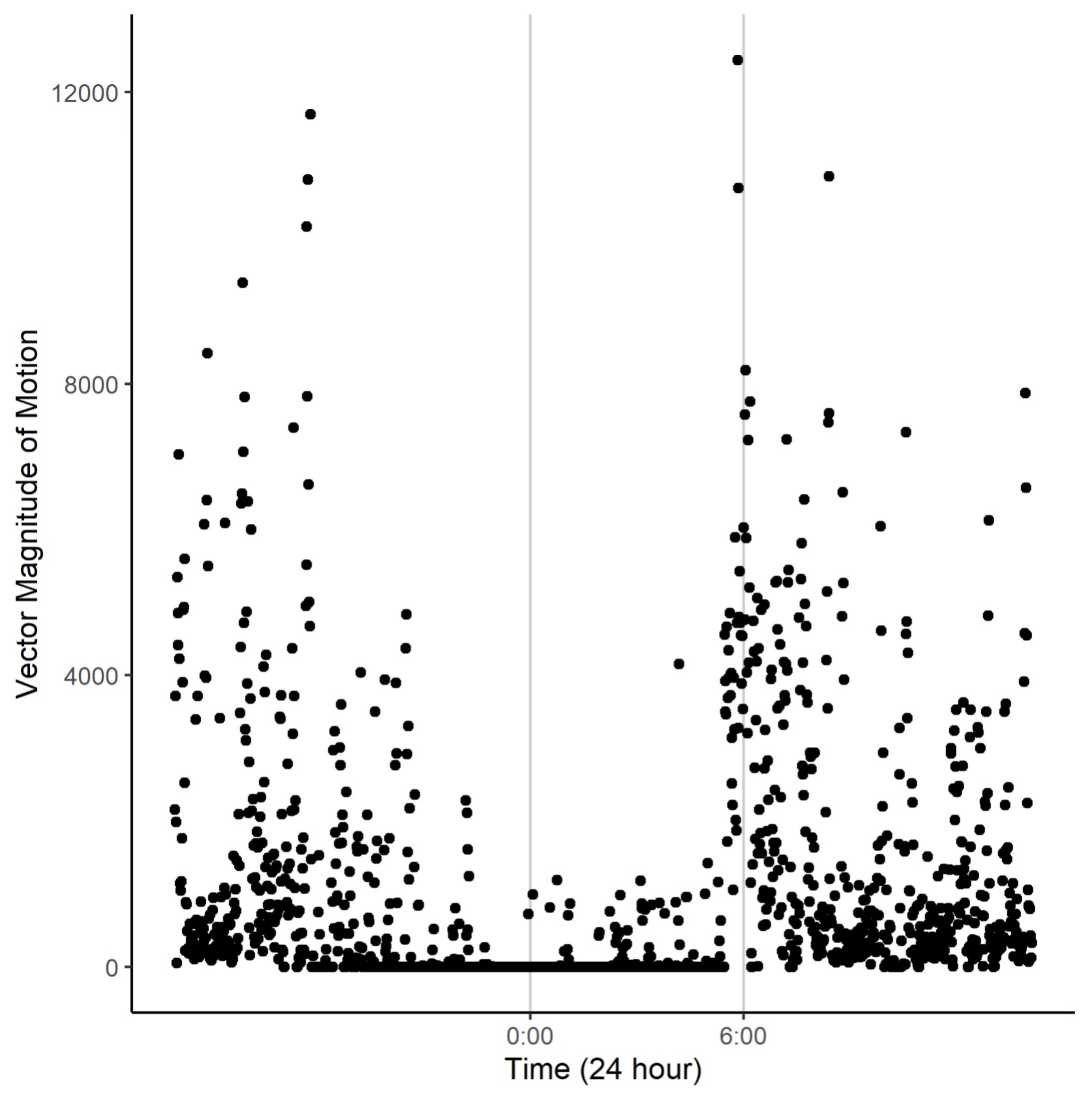

Supplement: S2 Fig — (TIFF) [file pdig.0000267.s005.tiff]

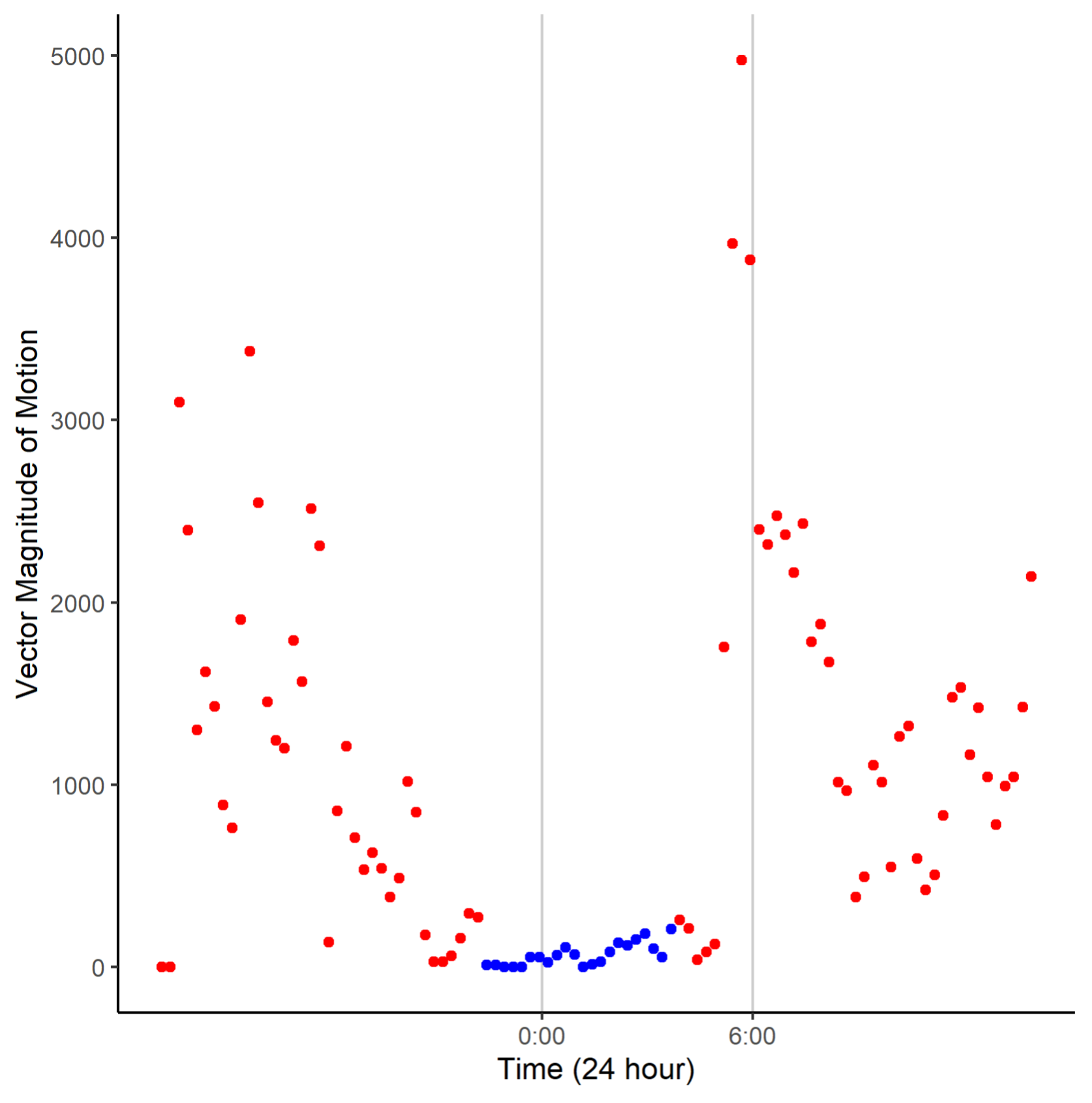

Supplement: S3 Fig — (TIFF) [file pdig.0000267.s006.tiff]

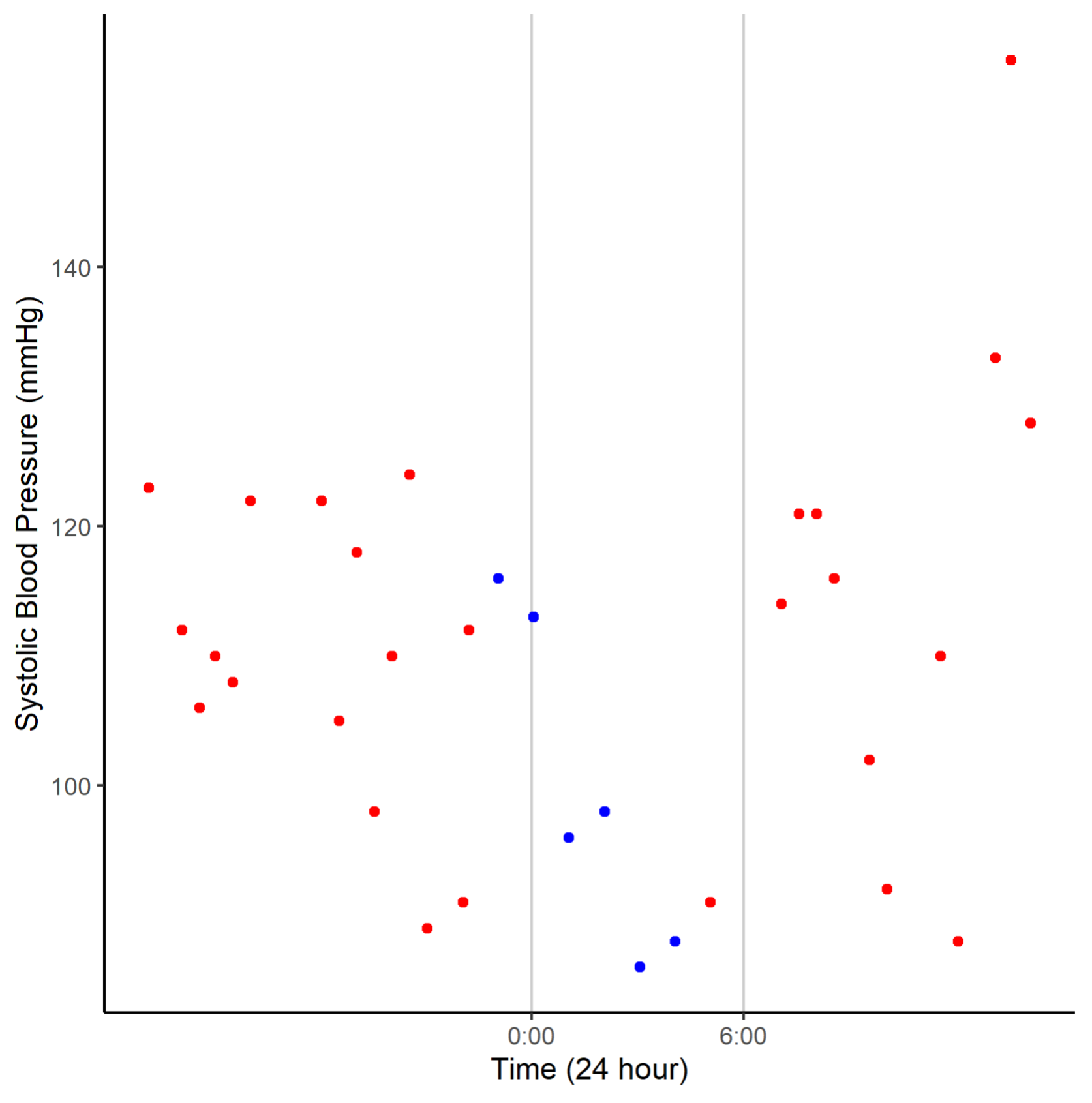

Supplement: S4 Fig — (TIFF) [file pdig.0000267.s007.tiff]

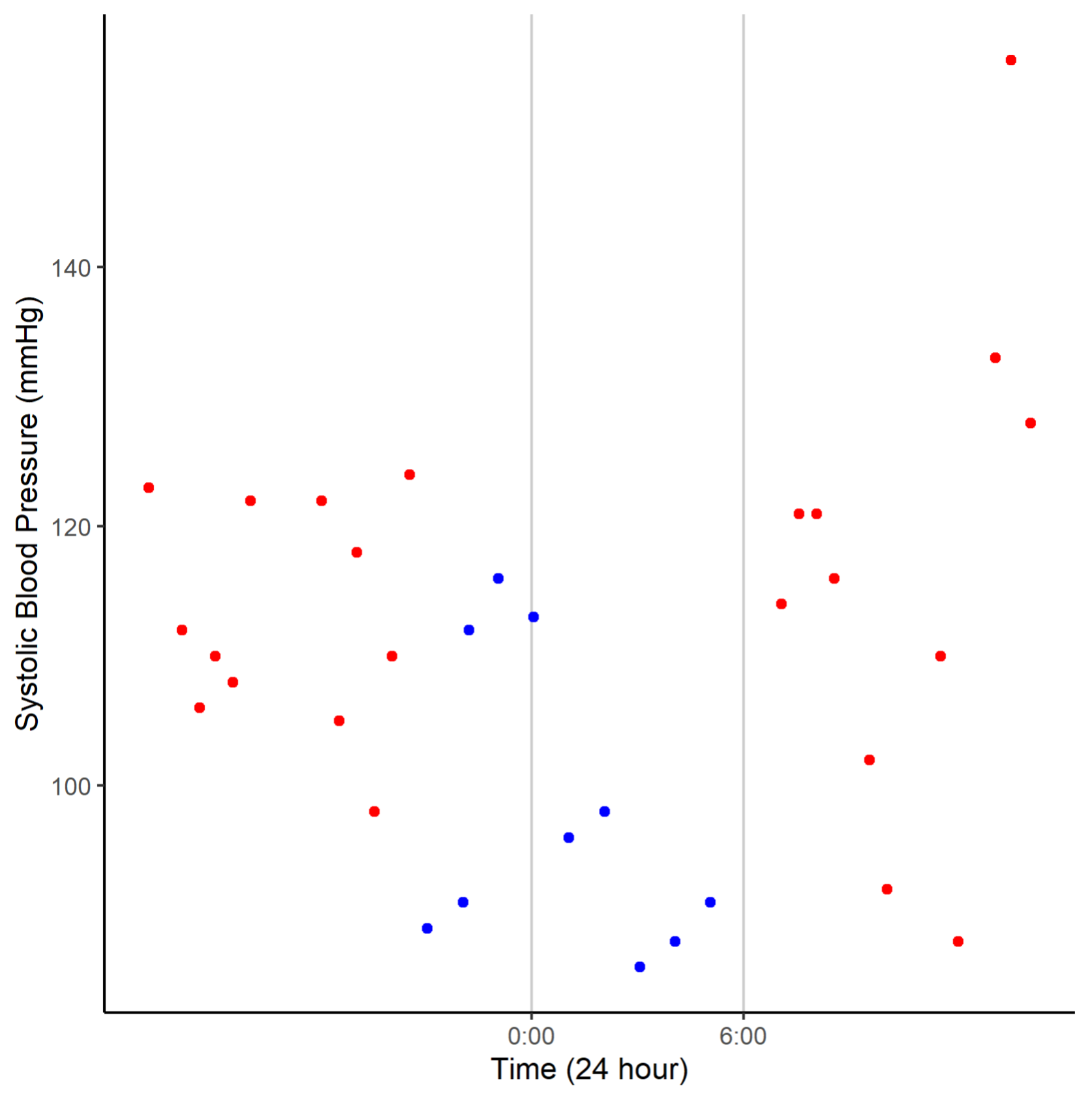

Supplement: S5 Fig — (TIFF) [file pdig.0000267.s008.tiff]

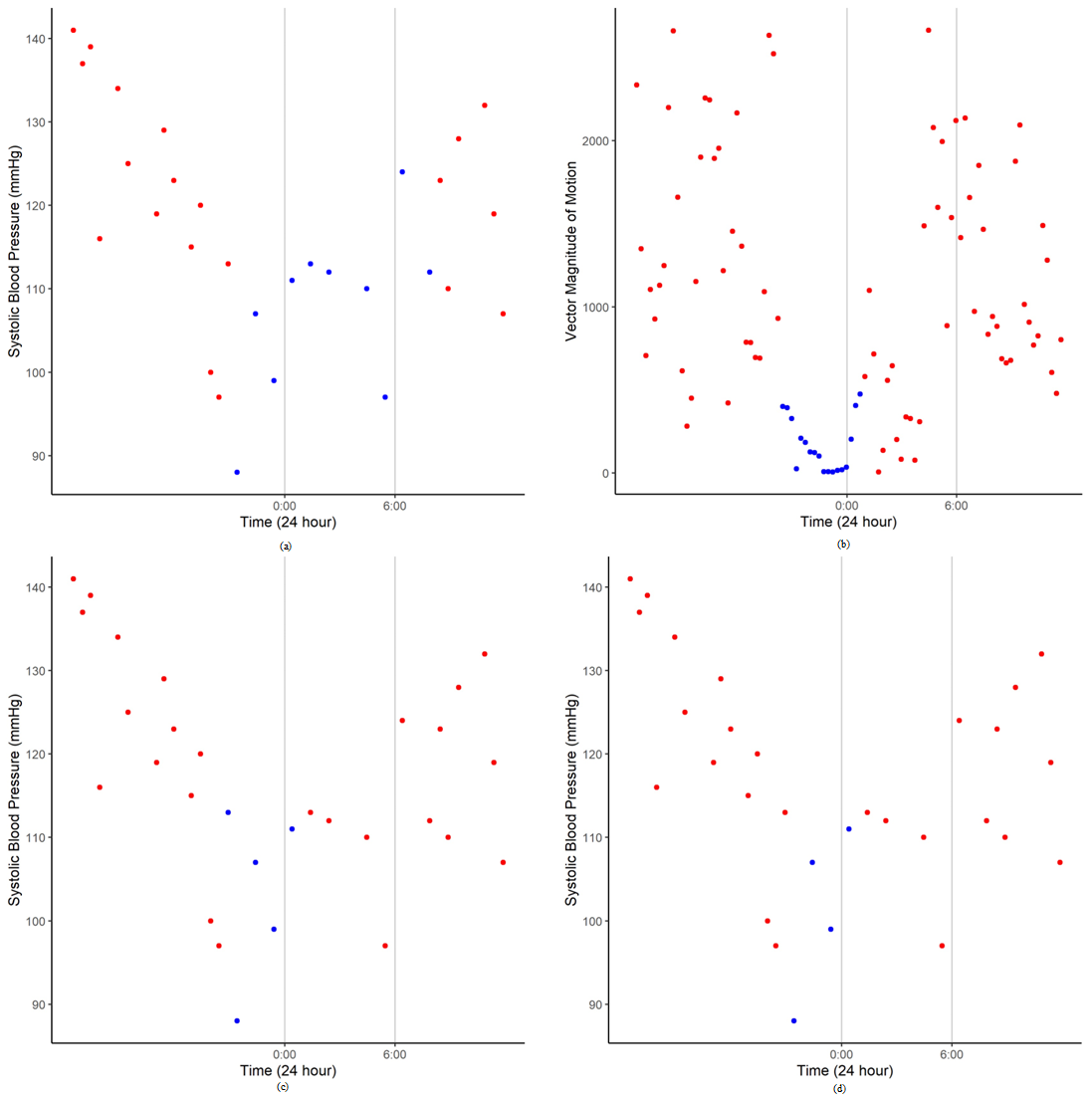

Supplement: S6 Fig — S6A Fig shows the ambulatory blood pressure monitoring data. S6B Fig shows the actigraphy analysis that shows the participant woke up, and while remained sedentary for a while after, was deemed to be awake and S6C Fig shows the recalculated nocturnal blood pressure with manual actigraphy analysis and S6D Fig with automated actigraphy analysis. (TIF) [file pdig.0000267.s009.tif]

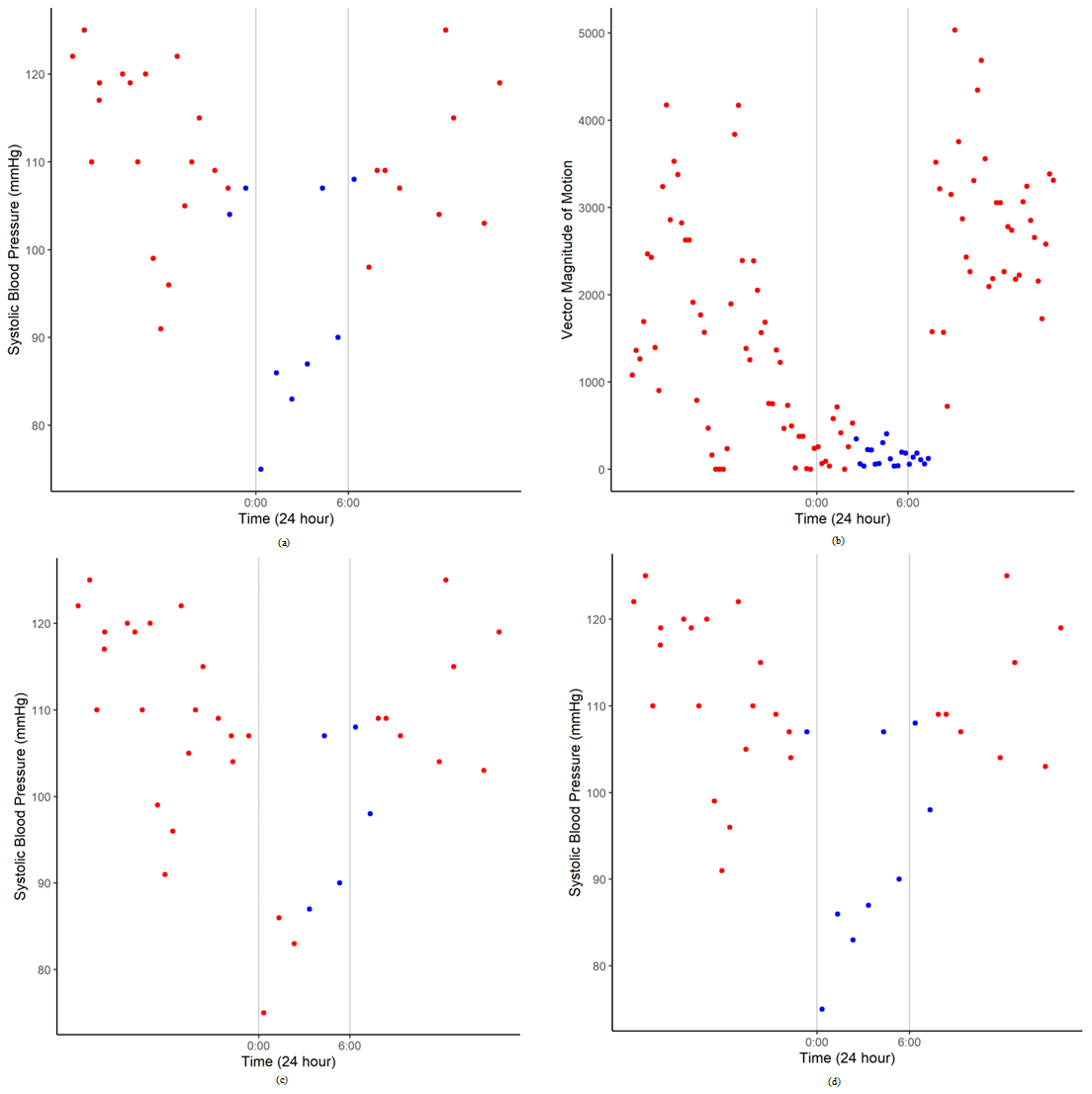

Supplement: S7 Fig — S7A Fig shows the ambulatory blood pressure monitoring data. S7B Fig shows the actigraphy analysis that shows the participant woke up, and while remained sedentary for a while after, was deemed to be awake, and S7C Fig shows the recalculated nocturnal blood pressure with manual actigraphy analysis and S7D Fig with automated actigraphy analysis. (TIF) [file pdig.0000267.s010.tif]
